# 2-Hydroxymelatonin Promotes Seed Germination by Increasing Reactive Oxygen Species Production and Gibberellin Synthesis in *Arabidopsis thaliana*

**DOI:** 10.3390/antiox11040737

**Published:** 2022-04-08

**Authors:** Hyoung Yool Lee, Kyoungwhan Back

**Affiliations:** Department of Biotechnology, College of Agriculture and Life Sciences, Chonnam National University, Gwangju 61186, Korea; xanthine@naver.com

**Keywords:** *Arabidopsis thaliana* (L.) Heynh. Columbia-0, dormant seed, gibberellin, germination, 2-hydroxymelatonin, melatonin, melatonin 2-hydroxylase, non-dormant seed, paclobutrazol, superoxide

## Abstract

It was recently reported that 2-hydroxymelatonin (2-OHM) is responsible for inducing reactive oxygen species (ROS) in plants. ROS are crucial molecules that promote germination through interaction with hormones such as gibberellic acid (GA). In this study, to confirm the pro-oxidant role of 2-OHM, we investigated its effect on seed germination in *Arabidopsis thaliana* (L.) Heynh. Columbia-0. We found that 2-OHM treatment stimulated seed germination by 90% and 330% in non-dormant and dormant seeds, respectively, whereas melatonin marginally increased germination (~13%) in both seed types compared to untreated control seeds. The germination promotion effects of exogenous 2-OHM treatment were due to increased ROS production followed by the induction of GA synthesis and expression of responsive genes. Accordingly, melatonin 2-hydroxylase (*M2H*), the gene responsible for 2-OHM synthesis, was strictly expressed only during the germination process. Further molecular genetic analyses using *m2h* knockout mutant and *M2H* overexpression clearly supported an increase in ROS triggered by 2-OHM, followed by increased expression of GA-related genes, which shortened the time to germination. Notably, 2-OHM application to *m2h* knockout mutant seeds fully recovered germination to levels comparable to that of the wild type, whereas melatonin treatment failed to increase germination. Together, these results indicate that 2-OHM is a pivotal molecule that triggers increased ROS production during seed germination, thereby enhancing germination via the GA pathway in *Arabidopsis thaliana*.

## 1. Introduction

In plants, melatonin acts as a multifunctional signaling molecule that displays a vast array of physiological functions in growth and defense responses in plants [1]. The major mechanism of these physiological roles of melatonin is closely coupled with its potent antioxidant activity, which regulates the cellular homeostasis of reactive oxygen species (ROS) in a spatial and temporal manner [2,3]. The signaling function of melatonin function may be conducted through a phytomelatonin receptor in a corresponding downstream signaling cascade that results in the induction of vast numbers of melatonin-responsive genes [4]. These melatonin reactions ultimately enhance protein quality control, metabolism modification, and plant defense tolerance against numerous environmental stresses encountered during plant growth and development [3,4,5,6,7,8,9].

Recent studies have discovered that melatonin is not an end product in plants, and that its various metabolites are not simple oxidation products of reactions between melatonin and ROS as observed in animals [10]. In plants, melatonin is enzymatically catalyzed into either cyclic 3-hydroxymelatonin (3-OHM) or 2-hydroxymelatonin (2-OHM) through reactions of the enzymes melatonin 3-hydroxylase (M3H) or melatonin 2-hydroxylase (M2H) [4]. The catalytic efficiency (*V*_max_/*K*_m_) of M3H and M2H is greater than that of melatonin biosynthesis enzymes such as *N*-acetylserotonin methyltransferase and caffeic acid *O*-methyltransferase [4]. Thus, 2-OHM is present in plants at concentrations that are hundreds of times higher than those of melatonin [11]. Unlike proposed inert catabolites [12,13], 2-OHM also induces defense genes [14] and is involved in plant tolerance to abiotic stresses [15] and cadmium [16]. Interestingly, 2-OHM has recently been reported to induce ROS production in a respiratory burst NADPH (nicotinamide adenine dinucleotide phosphate) oxidase (RBOH)-dependent manner, acting as a senescence-inducing factor in *Arabidopsis thaliana* [17].

ROS play a dual role as signaling molecules and cell death-inducing factors in all living organisms, including animals and plants [18,19]. Among their many functions, ROS play a key role in promoting germination, although their excessive accumulation in response to various stresses can also hinder germination [20]. For example, ROS increase in concentration during seed imbibition and play a positive role in seed germination, whereas seed germination was severely decreased in an *rbohD* mutant [21]. The key regulatory plant hormones gibberellic acid (GA) and abscisic acid (ABA) promote and inhibit germination, respectively; their balance controls germination, as GA cannot promote germination at high ABA concentrations [20,22]. Increased ROS levels during seed imbibition accelerate ABA catabolism and GA biosynthesis, resulting in enhanced seed germination [22]. However, important questions remain regarding the mechanisms of ROS production in seed imbibition and germination.

In this study, we compared the effects of 2-OHM and melatonin on seed germination in relation to ROS production. We further explored the expression of *M2H*, the key gene responsible for 2-OHM synthesis, during seed imbibition and germination. The results of this study will contribute to our understanding of the mechanisms by which 2-OHM is involved in seed germination in plants.

## 2. Materials and Methods

### 2.1. Plant Materials

*Arabidopsis thaliana* (L.) Heynh. Columbia-0 seeds (WT) were used in this study. The plants were grown under a 14 h light/10 h dark cycle at 25 °C, 60% humidity, and a photon flux density of 50 μmol m^−2^ s^−1^. The *m2h* knockout mutant (CS365315) generated by GABI Kat [23] and WT seeds were obtained from the Arabidopsis Biological Resource Center (ABRC; Ohio State University, Columbus, OH, USA) and the *m2h* knockout mutant was characterized by a *T-DNA* insertion in the *M2H* gene (AT3g60290) in the first intron. To confirm the mutant line as homozygous, polymerase chain reaction (PCR) screening was conducted using genomic DNA of the *m2h* knockout mutant line with the gene-specific primers 5′-GGA GGA AAC AAA TAA GAG TGT GG-3′ and 5′-AAG GCC ACA AAT TGA TCC AG-3′. 

### 2.2. Generation of Melatonin 2-Hydroxylase (M2H)-Overexpression A. thaliana Plant

To generate *M2H*-overexpressing transgenic *A. thaliana* plants, a full-length *M2H* cDNA sequence was amplified by reverse transcription (RT)-PCR using the primers 5′-AAA AAG CAG GCT ATG GAG GAA ACA AAT AAG-3′ and 5′-AGA AAG CTG GGT TCA GAG GTT TTT CTT TCT-3′. The promoter region of *M2H* gene sequences beginning 1261 bp upstream of the ATG start codon of the *M2H* gene was amplified by PCR from *A. thaliana* genomic DNA using the primers 5′-AAA AAG CAG GCT GGA GTT ATA TAG AGA TGC-3′ and 5′-AGA AAG CTG GGT TGT TTT TTT GCA AGA AAT-3′. Both *M2H* open reading frame (ORF) and promoter sequences were independently recombined into the pDONOR 221/Zeo vector using the BP reaction (Invitrogen, Carlsbad, CA, USA). For overexpression, pDONOR 221/Zeo-M2H was recombined into the plant transformation binary vector pYY63 [24], which harbors a cauliflower mosaic virus *35S* promoter through the LR reaction (Invitrogen). The pDONOR 221/Zeo-M2H promoter sequence was recombined into the pGWB533 binary vector [25] harboring the reporter gene encoding β-glucuronidase (GUS). These resulting binary constructs were transferred to *Agrobacterium tumefaciens* strain GV3101 using the freeze–thaw method and transformed into *A. thaliana* (L.) Heynh. Columbia (WT) plants using the floral dip method [26]. Transformed plants of the second generation after transformation were used. 

### 2.3. Germination Assays

To minimize the effects of seed maturation degree, plants of each tested genotype were grown in different sections of the same pot, and seeds were harvested and stored under identical conditions for <1 year (non-dormant seeds) or 1 week (freshly harvested dormant seeds). We placed 50 seeds in multi-well Petri dishes (5 mm diameter; SPL Life Science Co., Pocheon, Korea) with 0.1 mL germination medium (2 mM MgCl_2_, 5 mM MES, pH 5.7) under a 14 h light/10 h dark cycle at 25 °C (non-stratified) and a photon flux density of 50 μmol m^−2^ s^−1^. Seeds were considered to have germinated when the radical emerged through the seed coat. Seeds were examined using a stereomicroscope (SZ51, Olympus, Tokyo, Japan) and photographed using a 5.0-megapixel microscope digital camera (HY500US, Photonics, Oxford, MA, USA). Photographs were magnified to examine radical extrusion through the testa rupture. We obtained 2-OHM from Toronto Research Chemicals (North York, ON, Canada). Other chemicals such as paclobutrazol (Sigma-Aldrich, St. Louis, MO, USA), norflurazon (Sigma-Aldrich), and GA_4+7_ (Duchefa Biochemi, Haarlem, The Netherlands) were used in germination analyses. Stock solutions of all chemicals (1 mM) were dissolved in 2 mM MES buffer (pH 5.6 in 5 mM MgCl_2_) and diluted with the same buffer used in germination tests. Data are reported as averages of at least three experiments ± standard deviation (SD). 

### 2.4. Nitrotetrazolium Blue Staining 

Superoxide (O_2_^−^) was detected via in situ histochemical staining using nitrotetrazolium blue (NBT). Seeds were immersed in a solution containing 0.1% NBT (10 mM MES, pH 6.8) for 1 day, and then stored in ethanol (96%). Superoxide anions were visualized as precipitates of dark blue insoluble formazan compounds.

### 2.5. β-Glucuronidase (GUS) Staining 

The β-glucuronidase (GUS) activity was measured as described previously [27] and used to analyze the expression of the *M2H* gene promoter. Seeds were placed in GUS staining solution containing 50 mM sodium phosphate (pH 7.0), 0.5 mM potassium ferricyanide, 0.5 mM potassium ferrocyanide, 10 mM EDTA, 0.1% Triton X-100, and 1 mM X-Gluc (Sigma-Aldrich), and then incubated at 37 °C for 24 h. The stained embryos were immersed in 70% ethanol for several hours, and then microscopic analyses were conducted using an SZ51 stereomicroscope (Olympus).

### 2.6. RNA Analysis

Total RNA was extracted from seeds and leaves using the RNeasy Power Plant Kit (Qiagen, Tokyo, Japan) and Nucleospin RNA plant kit (Macherey-Nagel, Düren, Germany), respectively. The RNA (300 ng) was used to prepare cDNA using the RNA to cDNA EcoDry Premix system (Takara Clontech, Shiga, Japan). The 30 μL semiquantitative reverse transcription (RT)-PCR mixtures consisted of 1 μL cDNA, 0.1 μL of Ex Tag polymerase (TaKaRa Bio, Shiga, Japan), 3 μL of 10× Ex tag buffer system, 0.2 mM of each dNTP, and 2 mM MgCl_2_ and 0.4 pM each of the forward and reverse primers. The resulting PCR products were size-fractionated by electrophoresis in an 1% agarose gel and photographed. Expression levels of elongation factor-1 alpha (*EF-1α*; At5g60390) and *18s rRNA* (At1g80340) were used as loading controls for leaves and seeds, respectively. Real-time quantitative reverse transcription-PCR (qRT-PCR) analysis was performed on a Mic qPCR Cycler System (Bio Molecular Systems, Queensland, Australia) using SYBR Green RT-PCR Reagent Kit (Luna Universal qPCR Master Mix; NEB, Hitchin, UK) according to the manufacturer’s protocol. The relative expression levels were analyzed using Mic’s RQ software (Biomolecular Systems) and normalized to *18s rRNA*. The primer sequences for RNA analysis were as follows: *KS* (forward 5′-CCA AGT TGA TCT GGC AGG TA-3′; reverse 5′-TTG TCT CCT AAA ATC AAT TTT CCT C-3′), *GA3ox2* (forward 5′-CCC ATC TCT CAC TTG GAA ACA-3′; reverse, 5′-TTG TGA ATT TGA AGA ACC ACT CA-3′), *MYB33* (forward 5′-TTG TTC TTG GAG CAA CAT GC-3′; reverse 5′-TGC ATT GGC AGT TGC TAG TC-3′), *EXP2* (forward 5′- AGA CTT TTG AAG GCG GAC AA-3′; reverse 5′-AAG CGA GGA TAT GCG AAG AA-3′), *M2H* (forward 5′-GGA GGA AAC AAA TAA GAG TGT GG-3′, reverse 5′- TGC ATG CAA CTA GGT CCA AA-3′), *EF-1α* (forward 5′-TGG TGA CGC TGG TAT GGT TA-3′; reverse 5′-CAT CAT TTG GCA CCC TTC TT-3′), and *18s rRNA* (forward 5′-ATG ATA ACT CGA CGG ATC C-3′; reverse 5′-CCT CCA ATG GAT CCT CGT TA-3′). 

### 2.7. Statistical Analyses

Analysis of variance (ANOVA) was performed using IBM SPSS Statistics 25 software (IBM Corp. Armonk, NY, USA). Means with significant differences were identified using Tukey’s honest significant difference (HSD) post hoc tests, at a level of *p* < 0.05. Data are presented as means ± SD.

## 3. Results

### 3.1. Effects of Melatonin and 2-Hydroxymelatonin on A. thaliana Wild-Type Seed Germination

**Figure 1:**
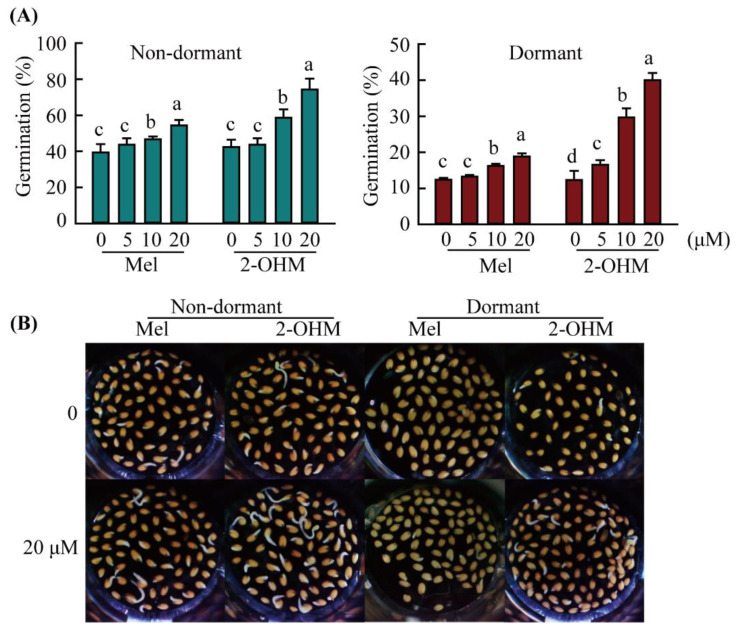
Effects of melatonin or 2-hydroxymelatonin (2-OHM) on *Arabidopsis thaliana* seed germination. (**A**) Effects of a range of melatonin and 2-OHM concentrations on the germination of non-dormant and dormant *A*. *thaliana* WT seeds. (**B**) Representative photographs of the effects of melatonin (Mel; 20 μM) or 2-OHM (20 μM) treatment on the germination of non-dormant and dormant *A*. *thaliana* WT seeds. Seeds were briefly sterilized and placed in multi-well plates with various concentrations of melatonin or 2-OHM solution (0.1 mL) prepared in 2 mM MgCl_2_ in 5 mM MES, pH 5.7 under light; germination percentages were evaluated at 40 h. The control solution (0 µM 2-OHM) contained 2 mM MgCl_2_ (in 5 mM MES, pH 5.7). Data are means ± standard deviations (SDs) of three replicates, each containing 50 seeds. Different letters denote significant differences (*p* < 0.05; analysis of variance (ANOVA), followed by Tukey’s honest significant difference (HSD) post hoc tests). Dormant seeds were freshly harvested seeds; non-dormant seeds were stored at 20 °C for at least 5 weeks.

The germination percentages of non-dormant (<1 year) and dormant (1-week-old) *A*. *thaliana* wild-type (WT) seeds were investigated at 40 h under light at 25 °C (Figure 1). The germination percentages of non-dormant and dormant wild-type (WT) were 40 and 12, respectively (*R*^2^ = 0.97). Melatonin (5 µM) treatment did not promote germination percentages in either non-dormant or dormant seeds, whereas 2-OHM treatment (5 μM) dramatically increased germination percentages in dormant seeds, but not in non-dormant seeds. Melatonin (20 µM) treatment increased germination percentages by ~13% on average in both non-dormant and dormant seeds, whereas the same concentration of 2-OHM increased germination percentages to 80 and 40 in non-dormant and dormant seeds, respectively. Notably, 2-OHM treatment significantly enhanced the germination percentages of dormant seeds in a dose-dependent manner compared to non-dormant seeds. At 20 µM concentration, 2-OHM treatment increased germination percentages by 0.4- and 2-fold compared to melatonin treatment in non-dormant and dormant seeds, respectively, indicating that 2-OHM, rather than melatonin, is a major compound promoting *A*. *thaliana* seed germination. In addition, the germination promotion effects of 2-OHM were much higher in dormant seeds than in non-dormant seeds.

### 3.2. Upregulated Expression of Gibberellic Acid (GA)-Related Genes by 2-Hydroxymelatonin (2-OHM) Treatment in Non-Dormant A. thaliana Seeds

**Figure 2:**
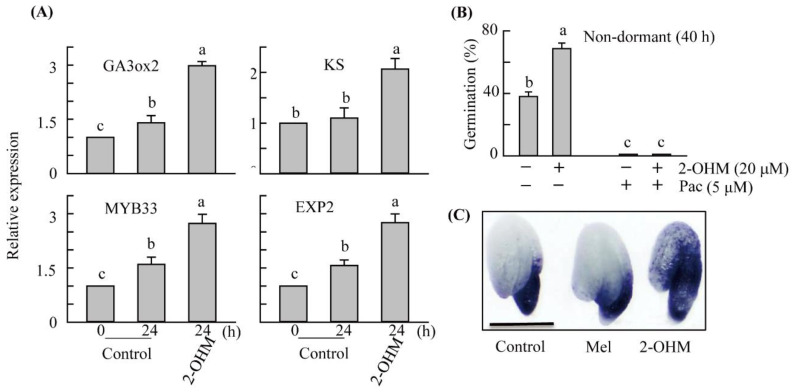
(**A**) Effects of 2-hydroxymelatonin (2-OHM) on the expression of gibberellic acid (GA)-related genes, (**B**) the GA biosynthesis inhibitor paclobutrazol, and (**C**) superoxide levels during seed imbibition. Non-dormant *Arabisopsis thaliana* wild-type (WT) seeds were imbibed in 2 mM MgCl_2_ (5 mM MES, pH 5.7) (Control) with or without 2-OHM (20 μM) for 1 day. Gene expression levels were analyzed by quantitative reverse-transcription polymerase chain reaction (qRT-PCR). Paclobutrazol (5 μM) was applied to determine whether 2-OHM-induced seed germination enhancement was dependent on GA. The germination percentage was determined at 40 h after imbibition. Nitrotetrazolium blue (NBT) staining was conducted to assess O_2_^.−^ accumulation. Bar = 0.3 mm. Error bars indicate standard deviation (SD) of three biological replicates. Different letters indicate significant differences (*p* < 0.05; ANOVA, followed by Tukey’s HSD post hoc tests). Pac, paclobutrazol; Control, 2 mM MgCl_2_; Mel, melatonin (20 μM); 2-OHM, 2-hydroxymelatonin (20 μM). *GA3ox2*, *gibberellin 3-oxidase 2* (AT1g80340); *MYB33*, *myb family transcription factor 33* (AT5g06100); *KS*, *ent-kaurene synthase* (AT1g79460); *EXP2*, *expansin 2* (AT5g05290); *18s rRNA* (AT1g80340).

GA and ROS play essential roles in promoting seed germination. To examine the effects of 2-OHM on GA and ROS, we applied 20 µM 2-OHM to non-dormant *A*. *thaliana* WT seeds for 40 h and evaluated the expression patterns of GA-related genes. The results showed that 2-OHM treatment greatly enhanced GA biosynthetic genes such as *gibberellin 3-oxidase 2* (*GA3ox2*) and *ent-kaurene synthase* (*KS*) compared to the mock control (Figure 2A). The expression levels of GA-responsive myb family transcription factor 33 (*MYB33*) and GA-responsive *Expansin 2* (*EXP2*) were significantly increased by 2-OHM treatment. Germination was completely abolished in both control and 2-OHM treatment seeds following treatment with paclobutrazol, a GA synthesis inhibitor (Figure 2B). These data clearly show that GA is an indispensable hormone in seed germination and is closely associated with 2-OHM-mediated promotion of seed germination. A previous study reported that 2-OHM treatment induced ROS production in aged leaves, but not young mature leaves, of *A*. *thaliana* [17]. To determine whether 2-OHM could induce ROS synthesis in *A*. *thaliana* seeds, we visualized superoxide levels through nitrotetrazolium blue (NBT) staining. *A*. *thaliana* seeds treated with 2-OHM (20 μM) showed denser NBT staining than those in the control and melatonin treatments (Figure 2C). These data clearly suggest that 2-OHM induces ROS production in *A*. *thaliana* seeds in a similar manner as shown in aged *A*. *thaliana* leaves [17]. 

### 3.3. Spatial and Temporal Expression of Melatonin 2-Hydroxylase (M2H) during Germination

**Figure 3:**
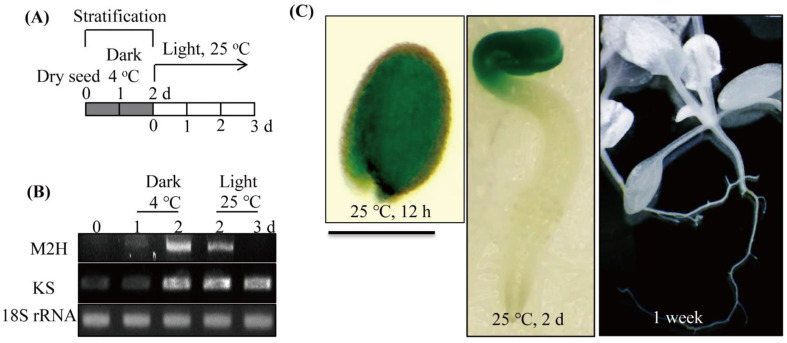
Melatonin 2-hydroxylase (*M2H*) expression in non-dormant *Arabidopsis thaliana* WT seeds during stratification and germination. (**A**) Schematic diagram of stratification and germination procedures. (**B**) RT-PCR analysis results for *M2H* and *KS* in WT seeds. (**C**) In situ β-glucuronidase (GUS) expression patterns at various stages of germination and in 1-week-old seedlings. WT seeds were imbibed at 4 °C in the dark for 2 days, and then transferred into light conditions at 25 °C. The pGWB533-M2H promoter:GUS transgenic line was used for GUS staining. The seed coat was removed for photograph after GUS staining on the embryo at 12 h under light incubation. Bar = 0.3 mm. GenBank accession numbers were as follows: *M2H*, *melatonin 2-hydroxylase* (At3g60290); *KS*, *ent-kaurene synthase* (AT1g79460); *18s rRNA* (AT1g80340).

*M2H* is the gene responsible for 2-OHM synthesis. To determine whether *A*. *thaliana M2H* (At3g60290) is specifically expressed during seed germination, we monitored *A*. *thaliana M2H* gene expression. *M2H* mRNA was not detected in dry seeds, whereas its expression was greatly expressed during stratification at 4 °C in the dark, and declined at 25 °C in light; after 3 days under the latter conditions, no *M2H* mRNA was observed (Figure 3). By contrast, *KS* showed peak expression at 2 days after stratification and maintained peak levels until 3 days of 25 °C in light. To confirm this transient and temporal expression of *M2H* mRNA, we generated *A*. *thaliana* transgenic lines expressing *M2H* promoter–GUS fusion. After 12 h at 25 °C, entire uncoated *A*. *thaliana* seeds showed strong GUS expression (Figure 3C), whereas after 2 days at 25 °C, GUS expression was observed only in the cotyledon, not in the hypocotyl, of germinated seeds. No GUS expression was observed in 1-week-old seedlings. These results together with *M2H* mRNA expression results indicate that *M2H* is transiently expressed in embryo tissues during seed germination, demonstrating that 2-OHM-mediated ROS production is highly specific to germinating seeds. 

### 3.4. Genetic Evidence for the Involvement of Melatonin 2-Hydroxylase (M2H) in Seed Germination 

**Figure 4:**
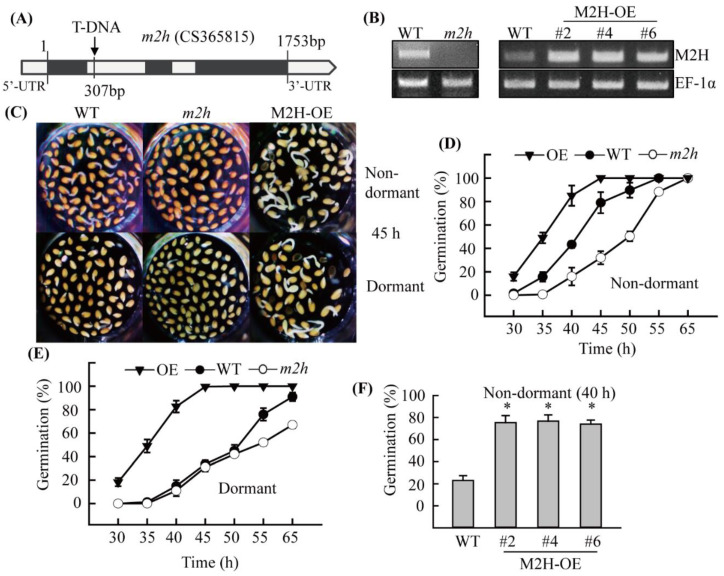
Germination results for *m2h* knockout mutant and *M2H* overexpression *A*. *thaliana* seeds. (**A**) Diagram of T-DNA insertion into *M2H* knockout *A*. *thaliana* (*m2h*). Black and white boxes indicate introns and exons, respectively. Arrows indicate the location of T-DNA insertion (not to scale). (**B**) Expression levels of *M2H* by RT-PCR in 7-week-old WT, *m2h knockout*, and *M2H* overexpression (M2H-OE) plants. Radical protrusion was photographed (**C**) at 45 h, and the germination ratio was quantified at various incubation times in (**D**) non-dormant seeds and (**E**) dormant seeds. (**F**) Seed germination in M2H-OE lines at 40 h after incubation. Seeds of WT, *m2h* knockout, and M2H-OE plants were germinated in solution containing 2 mM MgCl_2_ (5 mM MES, pH 5.7) in a growth room (25 °C). Seeds from three T_3_ homozygous plants of the M2H-OE transgenic lines were analyzed. Line #4 of M2H-OE was used in (**D**,**E**). Asterisks indicate significant differences from the wild type as determined by Tukey’s post hoc HSD test (*p* < 0.05). GenBank accession numbers were as follows: *M2H*, *melatonin 2-hydroxylase* (AT3g60290); *EF-1α*, *elongation factor-1 alpha* (AT5g60390). *EF-1α* was used as a loading control.

**Figure 5:**
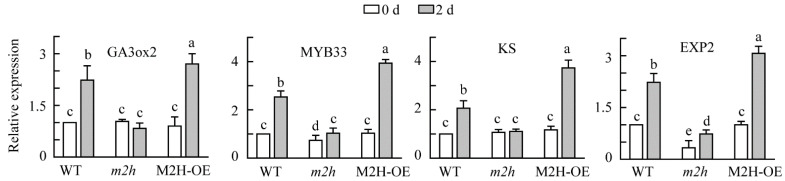
Reverse transcription quantitative real-time PCR (qRT-PCR) analysis results for genes involved in germination in various *Arabidopsis thaliana* lines. The relative expressions of *GA3ox2*, *KS*, *MYB33*, and *EXP2* in non-dormant WT, *m2h* knockout, and M2H-OE seeds were measured at 0 and 2 days after imbibition. Expression levels were normalized to *18s rRNA*. Different letters indicate significant differences (*p* < 0.05; ANOVA, followed by Tukey’s HSD post hoc tests). GenBank accession numbers were as follows: *GA3ox2*, AT1g80340; *MYB33*, AT5g06100; *KS*, AT1g79460; *EXP2*, AT5g05290; *18s rRNA*, AT1g80340. WT, *Arabidopsis thaliana* (L.) Heynh. Columbia-0; *m2h*, *M2H* knockout *Arabidopsis thaliana* line (CS365815); M2H-OE, *M2H* overexpression *Arabidopsis thaliana* line.

To obtain genetic evidence that *M2H* is required in seed germination, we selected an *m2h* knockout mutant (CS365815) carrying a T-DNA insertion in the first intron of *M2H* gene, which completely suppresses *M2H* expression (Figure 4A). We also generated *M2H* overexpression transgenic (M2H-OE) lines (Figure 4B). We performed a seed germination assay using *A*. *thaliana* seeds of the homozygous *m2h* knockout mutant and M2H-OE lines (Figure 4C–E). All three M2H-OE lines equally promoted germination at 40 h and 25 °C (Figure 4F). Non-dormant seeds of the M2H-OE line germinated much earlier than those of the WT, whereas *m2h* knockout mutant seeds showed significantly delayed germination compared to the WT (Figure 4D). However, all genotypes reached 100% germination by 65 h. Dormant M2H-OE seeds reached 100% germination at 45 h, whereas WT reached 100% germination at 65 h. However, the *m2h* knockout mutant showed 60% seed germination at 65 h (Figure 4E). These data suggest that *M2H* is required for seed germination in *A*. *thaliana*. To determine whether enhanced germination in the M2H-OE line and decreased germination in the *m2h* knockout mutant are associated with the expression of germination-related genes, we quantified mRNA levels at 2 days of 25 °C in light (Figure 5). Four genes (*GA3ox2*, *KS*, *MYB33*, and *EXP2*) were induced at 2 days, compared to 0 days for dry WT *A*. *thaliana* seeds. However, these genes were not induced in *m2h* knockout mutant seeds at 2 days, whereas M2H-OE seeds showed much higher levels of these four genes than the WT. Together, these results indicate that *M2H*, the key gene for 2-OHM synthesis, plays an important role in *A*. *thaliana* seed germination via 2-OHM synthesis.

### 3.5. Gibberellic Acid (GA)- and Abscisic Acid (ABA)-Dependent Germination in WT, m2h Knockout, and M2H-OE Lines

**Figure 6:**
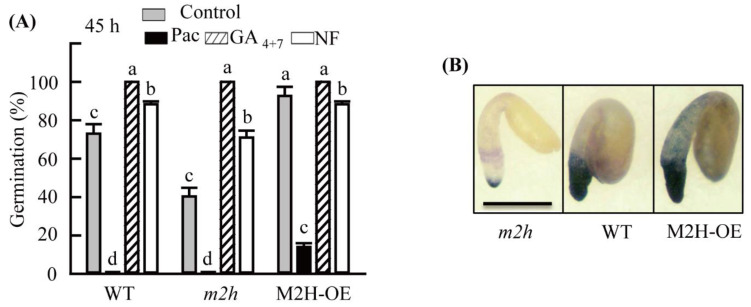
Effects of gibberellic acid (GA), GA inhibitor (paclobutrazol), and abscisic acid (ABA) inhibitor (norflurazon) on seed germination in various *A*. *thaliana* lines. (**A**) Percentage of germination upon various chemical treatments at 45 h after imbibition. (**B**) Levels of superoxide radical (O_2_^−^) determined by nitrotetrazolium blue (NBT) staining in WT, *m2h* knockout mutant, and M2H-OE seeds at 24 h after imbibition. Seeds were germinated in control solution (2 mM MgCl_2_ in 5 mM MES, pH 5.7) with or without various chemicals. Bar = 0.3 mm. GA, gibberellic acid; *m2h*, melatonin 2-hydroxylase knockout mutant line; M2H-OE, melatonin 2-hydroxylase overexpression line; NF, norflurazon; Pac, paclobutrazol. Different letters indicate significant differences among lines (*p* < 0.05; ANOVA, followed by Tukey’s HSD post hoc tests).

Paclobutrazol (5 µM) treatment completely abolished seed germination in non-dormant seeds of both WT and *m2h* knockout mutant at 45 h in light, whereas M2H-OE showed 10% seed germination (Figure 6). GA (5 µM) treatment promoted seed germination to 100% in both WT and *m2h* knockout mutant seeds. The ABA synthesis inhibitor norflurazon also enhanced seed germination in both the WT and *m2h* knockout mutant; however, its enhancement was lower than that of the GA treatment. These results indicate that delayed seed germination in the *m2h* knockout mutant is mainly attributable to GA levels, although ABA is also involved in seed germination to a lesser degree. Superoxide radical testing showed stronger nitrotetrazolium blue (NBT) staining in the M2H-OE line than in the WT, suggesting higher ROS production in M2H-OE seeds than in WT seeds. By contrast, the *m2h* knockout seeds showed much weaker staining that WT seeds. These data clearly show that *M2H* is closely involved in 2-OHM synthesis in *A*. *thaliana* seed. 

### 3.6. Recovery of Seed Germination by 2-Hydroxymelatonin (2-OHM) Treatment in m2h Knockout Mutant Seeds

**Figure 7:**
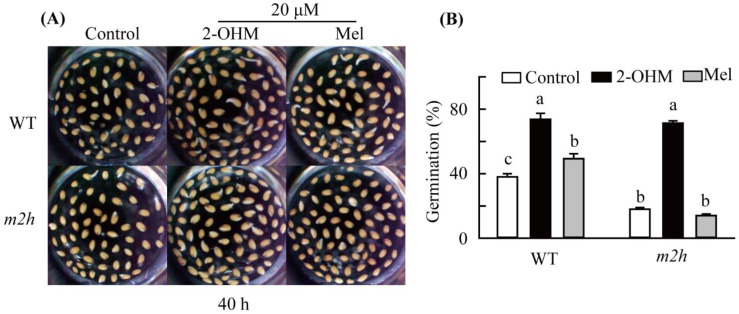
Gain-of-function analysis of *m2h* knockout mutant seed germination following exogenous 2-hydroxymelatonin (2-OHM) treatment. Non-dormant *A. thaliana* WT and *m2h* knockout mutant seeds were germinated with 2-OHM (20 μM) or without 2-OHM (Control) at 25 °C for 40 h. (**A**) Photographs of germinated seeds upon various chemical treatments at 40 h after imbibition. (**B**) Quantification of germination in (**A**). Error bars indicate standard deviation (SD) of three biological replicates. Different letters indicate significant differences (*p* < 0.05; ANOVA, followed by Tukey’s HSD post hoc tests).

To demonstrate the involvement of 2-OHM in *A*. *thaliana* seed germination, *m2h* knockout mutant seeds were treated with 2-OHM to determine whether seed germination capacity would recover to that of WT seeds. We found that 2-OHM treatment (20 μM) promoted seed germination percentages from 40% to 80% in non-dormant *A*. *thaliana* WT seeds (Figure 7). Similarly, the increase in seed germination percentages relative to the WT increased from 20% to 80% in the *m2h* knockout mutant following treatment with 20 µM 2-OHM. By contrast, melatonin treatment did not promote seed germination in *m2h* knockout mutant seeds, suggesting a slight promotional effect of melatonin in WT seeds caused by 2-OHM, which is synthesized from melatonin through abundant *M2H* expression during seed germination. 

## 4. Discussion

Seed germination undergoes multiple complex physiological processes requiring a series of hormones and signaling molecules, and a vast array of gene expression patterns [28]. Two important signaling molecules, nitric oxide (NO) and hydrogen peroxide (H_2_O_2_), work together to induce seed germination. Generally, H_2_O_2_ treatment promotes seed germination in concert with NO increase [22,29]. The primary role of H_2_O_2_ in seed germination is the direct regulation of gibberellic acid (GA) biosynthesis and indirect regulation of abscisic acid (ABA) catabolism. Consistent with the promotional roles of exogenous H_2_O_2_ treatment, H_2_O_2_ levels increase during seed imbibition and germination [21,22], and its endogenous increase in the catalase knockout mutant *cat2–1* showed dramatically increased seed germination, indicating the key positive role of ROS in seed germination [21]. These findings indicate that reactive oxygen species (ROS) are key signaling molecules acting upstream of GA and ABA [22]. However, no studies have reported the mechanism by which ROS are induced during germination. 

Melatonin has been observed in all living organisms, including animals, plants, and bacteria [30,31]. In plants, melatonin is involved in multiple processes, including seed germination [32,33], seedling growth [34,35], flowering [36,37], and yield increase [38,39]. The pleiotropic physiological role of melatonin in plants has recently been proposed to be controlled by a combination of melatonin and its metabolites such as 2-hydroxymelatonin (2-OHM) [4] because 2-OHM also acts as an important signaling molecule, but plays a completely different role from the antioxidant role of melatonin [9]. For example, melatonin is a potent antioxidant [19], whereas 2-OHM is a pro-oxidant, as its treatment induces ROS in a respiratory burst NADPH oxidase (RBOH)-dependent manner in plants [17]. Interestingly, melatonin was rapidly converted into 2-OHM by the action of M2H enzymes in rice seedlings [40,41]. Because two contrasting molecules (melatonin and 2-OHM) are present or synthesized when plants are challenged with melatonin, caution is required in the interpretation of analysis results. Because *M2H* is strictly regulated at the tissue level according to circadian rhythm and stress, 2-OHM synthesis upon melatonin treatment is also tightly regulated [42]. Although 2-OHM is involved in responses to stresses such as cadmium [16] and combined cold and drought [15], the ROS induction-related mode of action of 2-OHM was first examined in *A*. *thaliana* [17]. Therefore, we investigated further unknown functions of 2-OHM, focusing on a potential role for 2-OHM in seed germination, because ROS are crucial seed germination-promoting molecules in plants [43]. As observed in aged leaves [17], 2-OHM treatment enhanced ROS production in *A*. *thaliana* seeds, followed by a significant increase in germination percentages in both dormant and non-dormant *A*. *thaliana* seeds (Figure 1). Consequently, a number of genes encoding GA synthesis [36] and GA-responsive genes [44] were induced, whereas treatment with the GA synthesis inhibitor paclobutrazol nullified the 2-OHM-induced seed germination effect, indicating that 2-OHM-mediated germination enhancement is GA-dependent (Figure 2). These results demonstrating 2-OHM effects on seed germination were further confirmed by loss- and gain-of-function analyses using *m2h* knockout mutant and M2H-OE *A*. *thaliana* seeds (Figure 4, Figure 5 and Figure 6). *M2H* and GUS expression in vivo clearly suggested that 2-OHM is closely associated with seed germination (Figure 3). 

**Figure 8:**
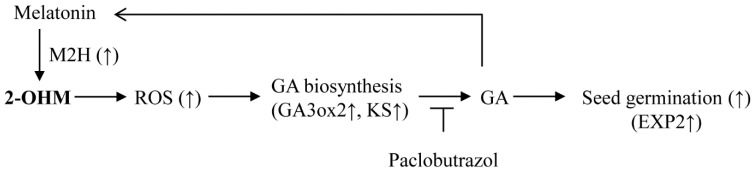
Schematic model showing 2-hydroxymelatonin (2-OHM)-mediated seed germination enhancement in *A*. *thaliana* seeds. Seed imbibition induced melatonin 2-hydroxylase (*M2H*) expression, leading to 2-OHM synthesis, which triggered reactive oxygen species (ROS) production followed by gibberellic acid (GA) biosynthesis, resulting in increased seed germination. Paclobutrazol treatment nullified 2-OHM-mediated seed germination enhancement. GA was positively involved in the synthesis of melatonin, which acts as a substrate for the M2H enzyme in 2-OHM biosynthesis. Upward arrows indicate upregulation. *GA3ox2*, *gibberellin 3-oxidase 2*; *KS*, ent-kaurene synthase; *EXP2*, *expansin 2*.

Therefore, we propose a potential model for the trigger of ROS production by 2-OHM synthesized by transient *M2H* expression during seed germination, followed by GA synthesis resulting in accelerated seed germination (Figure 8). Notably, GA acts as an endogenous elicitor of melatonin synthesis, providing a substrate for M2H enzymes in 2-OHM synthesis [45]. 

## 5. Conclusions

ROS play key signaling roles in the initiation of both senescence and seed germination [29,46]; however, little is known about the upstream signaling molecules that induce ROS. Although plant hormones such as ethylene and ABA also trigger ROS accumulation in plants, their specificity in ROS production is only weakly supported because they are involved in a wide range of biological processes. Unlike ethylene and ABA, 2-OHM does not act as a hormone but rather as a signaling molecule inducing ROS in a tissue-specific manner, as demonstrated in leaf senescence and seed germination. By contrast, melatonin is a potent antioxidant that has been proposed to regulate many biotic and abiotic stress responses through modulating ROS homeostasis [2]. The results of the present study indicate that melatonin acts as an antioxidant, whereas 2-OHM acts as a pro-oxidant; therefore, we hypothesize that the balance between melatonin and 2-OHM may orchestrate a wide range of biological processes ranging from seed germination to senescence and embryogenesis [2,4,47]. Although melatonin displays different gene expression profiles from 2-OHM [17], many previously reported physiological effects of melatonin must be reevaluated in the *m2h* knockout mutant, where the effects of 2-OHM are excluded. Further in-depth studies considering the action of 2-OHM will open a new avenue for melatonin research in plants. 

## Data Availability

The data presented in this study are available within the article.
